# Indirect exclusion of four candidate genes for generalized progressive retinal atrophy in several breeds of dogs

**DOI:** 10.1186/1477-5751-5-19

**Published:** 2006-11-29

**Authors:** Tanja Lippmann, Sandra M Pasternack, Britta Kraczyk, Sabine E Dudek, Gabriele Dekomien

**Affiliations:** 1 Human Genetics, Ruhr-University Bochum, Germany

## Abstract

**Background:**

Generalized progressive retinal atrophy (gPRA) is a hereditary ocular disorder with progressive photoreceptor degeneration in dogs. Four retina-specific genes, ATP binding cassette transporter retina (*ABCA*4), connexin 36 (*CX*36), c-mer tyrosin kinase receptor (*MERTK*) and photoreceptor cell retinol dehydrogenase (*RDH*12) were investigated in order to identify mutations leading to autosomal recessive (ar) gPRA in 29 breeds of dogs.

**Results:**

Mutation screening was performed initially by PCR and single strand conformation polymorphism (SSCP) analysis, representing a simple method with comparatively high reliability for identification of sequence variations in many samples. Conspicuous banding patterns were analyzed via sequence analyses in order to detect the underlying nucleotide variations. No pathogenetically relevant mutations were detected in the genes *ABCA*4, *CX*36, *MERTK *and *RDH*12 in 71 affected dogs of 29 breeds. Yet 30 new sequence variations were identified, both, in the coding regions and intronic sequences. Many of the sequence variations were in heterozygous state in affected dogs.

**Conclusion:**

Based on the ar transmittance of gPRA in the breeds investigated, informative sequence variations provide evidence allowing indirect exclusion of pathogenetic mutations in the genes *ABCA*4 (for 9 breeds), *CX*36 (for 12 breeds), *MERTK *(for all 29 breeds) and *RDH*12 (for 9 breeds).

## Background

Single strand conformation polymorphism (SSCP) analysis is a simple and cost-effective method with high reliability for the identification of base substitutions or other sequence variations like small deletions or insertions in large cohorts of individuals [1]. We used SSCP analyses in order to define genetic alterations causing generalized progressive retinal atrophy (gPRA) in dogs. gPRA, like retinitis pigmentosa (RP) in man, represents a genetically heterogeneous disorder [2]. Usually, gPRA starts with night blindness and continues with restrictions in the visual field. The final stage of disease is typically complete blindness. In different dog breeds, different ages at onset are observed as well as variable rates of disease progression [2,3]. Generally, gPRA is inherited in an autosomal recessive (ar) manner. Until now, causal mutations for ar gPRA have been identified exclusively in few dog breeds [3-7]. On the other hand, a number of photoreceptor genes have been excluded as the primary genetic cause of the trait in up to 26 dog breeds [8-15] investigated here.

In this study we investigated four retina-specific genes, ATP binding cassette transporter retina (*ABCA*4), connexin 36 (*CX*36), c-mer tyrosin kinase receptor (*MERTK*) and photoreceptor cell retinol dehydrogenase (*RDH*12) for mutations leading to ar gPRA in 29 breeds of dogs. The *ABCA*4 gene encodes the ABCR or RIM protein (RmP), thought to act as a flippase for N-retinylidene phosphatidylethanolamine (N-retinylidene-PE), thereby facilitating the transport of all-trans-retinal from the disk lumen to the photoreceptor cytoplasm [16,17]. Mutations in the *ABCA*4 gene have been associated with ar transmitted Stargardt disease (STGD1), ar inherited cone-rod dystrophy (CRD) and ar RP [18-25]. CX36 gap junction channels are responsible for distinct coupling patterns of ganglion cells in inter- and output-neurons of the retina [26] and are essential for normal synaptic transmission within the rod pathway. In CX36-deficient mice the b-wave of the electroretinogram is primarily affected, suggesting that CX36 is involved in the pathways generating the b-wave [27]. Furthermore, the *MERTK *gene encodes a receptor tyrosine kinase known as MER. The retinal pigment epithelium is the major site of *MERTK *expression in the retina. In a certain rat strain (Royal College of Surgeons, RCS) a small deletion in this gene leads to retinal degeneration [28]. Finally, RDH12 protein is involved in the production of 11-*cis*-retinal from 11-*cis*-retinol during regeneration of the cone visual pigments [29]. Mutations in the encoding gene *RDH*12 cause childhood-onset severe retinal dystrophy [30].

We describe here the mutation screening results in four canine retina-specific genes by SSCP analysis and DNA sequencing in 29 breeds of dogs. In addition, we use identified single nucleotide polymorphisms (SNP) for indirect exclusion of respective candidate genes in the analyzed pure-bred populations (based on the fact of 'founder effect' and inbreeding) for ar transmitted gPRA.

## Results and discussion

We analyzed 71 unrelated dogs with an established clinical diagnosis of gPRA as well as 13 unaffected controls. In order to detect mutations in the *ABCA*4, *CX*36, *MERTK *or *RDH*12 genes we performed pre-screening by SSCP of genomic DNA and DNA sequencing for all band shifts detected. No pathogenetically relevant mutations were identified in the analyzed breeds of dogs. But we observed several polymorphisms, both, in the coding regions and intronic sequences, in the analyzed DNAs as summarized in see Additional file 1. These DNA sequence variations can be used as intra-genic markers, thus excluding segregation with ar gPRA. According to the breeding history ('founder effect' base on small numbers of dogs that were used to start the breed) and small population size of most breeds investigated, exclusively a single disease causing mutation is expected per breed. Furthermore, breeding histories point to few meiotic events, in which intra-genic recombinations could have occurred between an unidentified mutation in the gene locus in gPRA dogs and the investigated polymorphism. Animals affected by an ar transmitted trait are expected to be typed homozygously not only for the disease causing mutation but also for tightly linked non-pathogenic DNA sequence variations in the same gene. For that reason a polymorphism in heterozygous state in an affected dog will practically exclude mutations in the analyzed candidate gene as being implicated in the retinal disorder in a defined breed [12,31].

The *ABCA*4 gene consists of 47 exons. In addition to exons 2, 3, 6, 9–10, 12–13, 21–22, 28–29, 34–35, 39–40 and 45–47, whenever possible the conserved splice sites were also screened for sequence variations. The other exons were not examined, because we have detected many informative sequence variations in the analyzed parts of the *ABCA*4 gene which allowed indirect gene analysis. Altogether, we identified 18 SNP, 5 within exons 6, 13 and 29 as well as 13 SNP within introns 6, 9, 21, 28, 45 and 46 [see Additional file 1]. The SNP were present in heterozygous state in affected dogs in 18 of the 24 investigated breeds. With respect to the abovementioned background, the *ABCA*4 gene represents an exceptional case, because the gene spans a large genomic region of 150 kb. Therefore, recombination events have to be taken into account for this gene between a hypothetical unidentified mutation and the investigated SNP. Consequently, indirect exclusion of this candidate gene is only possible for those breeds for which affected dogs are typed heterozygous for polymorphisms in the 5' and the 3' parts of the gene. Under this conservative precondition, we excluded mutation in the *ABCA*4 gene as a cause for gPRA in the breeds Berger des Pyrénées, Kucasz, Lowchens, Saarloos, Salukis, Scottish Terriers, Schapendoes, Sloughis and Tibetan Terriers.

During SSCP analysis of the 2 exons (and adjacent intron/exon boundaries) of the *CX*36 gene, two SNP were identified within the ORF, both not causing amino acid exchanges. The c.621T>C transversion was identified only in Afghan Hounds and Salukis; the c.678A>G variation occurs in 16 breeds [see Additional file 1]. In 11 of the 24 investigated breeds this sequence variation was found in heterozygous state in gPRA-affected dogs. Because of the small size of the gene and the above described reasoning, *CX*36 mutations can be excluded as the cause of gPRA within Airedale Terriers, Afghan Hound, Conton de Tulears, Dachshunds, Kuvasz, Lowchens, Miniature Poodles, Saarloos, Salukis, Scottish Terriers, Sloughis and Tibetan Terriers.

The *MERTK *gene was examined here for polymorphisms in 29 breeds by PCR-SSCP analysis of exons 4, 6–8 and 10 including splice donor and acceptor sites as well as adjacent intronic sequences. Because seven single base substitutions were identified in all analyzed exons as well as one SNP in intron 7, we did not investigate the *MERTK *gene any further. Our typing results suggest the sequence variations in the *MERTK *gene are not causative for gPRA in all 29 analyzed breeds [see Additional file 1].

In the seven exons of the *RDH*12 gene (including all intronic splice signal sequences), two sequence variations were obvious: an infrequent by observed sequence exchange in exon 1 and a more common SNP in intron 4 [see Additional file 1]. Since diseased animals were heterozygous for the polymorphism in intron 4, the *RDH*12 gene is very unlikely to harbor any critical mutation causing gPRA in the following breeds: Airedale Terriers, Conton de Tulears, Glen of Imaal Terriers, Giant Schnauzers, Golden Retrievers, Kuvasz, Lowchen, Miniature Poodles and Dachshunds.

In 16 of the investigated dog breeds exclusively one gPRA affected animal was available for mutation analysis [see Table 1]. For these breeds an exclusion of the investigated genes is not regarded as being definitive. Altogether, the mutation screening of the four genes performed by SSCP analysis revealed 30 new SNP. This fact underscores that SSCP screening is still a quite useful, sensitive [1] and cost-effective method as of today, especially for a large number of DNA samples. The newly identified SNP were used for exclusion of the four investigated candidate genes in canine eye disease for a large number of analyzed breeds. In addition, the identified SNP in the *ABCA*4, *CX*36, *MERTK *and *RDH*12 genes occurred in several breeds, rendering them useful markers for future studies.

**Table 1:** Dog breeds examined for gPRA causing mutations.

Breed (abbreviation)	Number of dogs investigated	gPRA-affected dogs
Afghan Hound (AW)	1	1
Airedale Terrier (AT)	6	5
Akita Inu (Aki)	2	1
Bearded Collie (BCo)*	2	2
Berger des Pyrénées (BDP)	2	1
Bichon Bolognese (Bo)	1	1
Conton de Tulear (CdT)	5	4
Curly-Coated Retriever (CCR)	1	1
Flat-Coated Retriever (FCR)*	1	1
Giant Schnauzer (GS)	1	1
Glen of Imaal Terrier (GIT)*	8	7
Golden Retriever (GR)	3	1
Jack Russel Terrier (JRT)	1	1
Kuvasz (Ku)	4	3
Lowchen (Lo)	7	6
Miniature Poodle (MP)	4	4
Newfoundlanddog (NF)	1	1
Polish Lowland Sheepdog (PON)	1	1
Rottweiler (Ro)	1	1
Saarloos/Wolfshound (Sa)	7	6
Saluki (Persian Greyhound; Sal)	2	1
Schapendoes (Dutch Sheepdog; SD)	5	4
Scottish Collie (Co)	2	2
Scottish Terrier (ScT)	1	1
Sloughi (Arabian Greyhound; Sl)	3	2
Standard Schnauzer (SS)*	1	1
Tibetan Terrier (TT)	4	4
Wire-haired Dachshund (WD)	6	6
Yorkshire Terrier (Y)*	1	1
Total	84	71

* Examined only for *MERTK *and *RDH12 *genes

## Conclusion

Mutation screening for gPRA in four canine retina-specific genes (*ABCA*4, *CX*36, *MERTK *and *RDH*12 gene) was performed by SSCP analysis and DNA sequencing. Even though no pathogenetically relevant mutations were detected in 71 affected dogs of 29 breeds, 30 new sequence variations were identified. These single nucleotide polymorphisms were subsequently used for indirect exclusion of the 4 candidate genes for autosomal recessively transmitted gPRA in the analyzed pure-bred populations (based on the fact of 'founder effect' and inbreeding). Using this approach, the indirect exclusion of pathogenetic mutations in the *ABCA*4 gene was possible for 9 breeds, in the *CX*36 gene for 12 breeds, in *MERTK *for all 29 breeds and in *RDH*12 for 9 breeds.

## Materials and methods

Blood from 84 dogs of 29 different breeds, including 71 gPRA-affected animals [see Table 1] was collected with the permission of the owner and in cooperation with breeding organizations. For two of these breeds the causative gPRA mutations are already known (Sloughi: [7]; Miniature Poodles: patented by OptiGen). These breeds were included as controls for the analysis of possibly found potential mutations.

In some cases a dominant inheritance of gPRA or misdiagnosis of gPRA due to phenocopies might be theoretically possible, but the performed pedigree analyses (data not shown) suggest that ar inheritance prevails on all accounts in the breeds analyzed here. The pedigree analyses also show, that the affected dogs of the breeds for which several samples were available were closely related. This could strengthen the assumption that within these breeds each gPRA affected dog has the same causal mutation. Experienced veterinarians confirmed the gPRA status of affected and unaffected dogs by ophthalmoscopy as documented in certificates of the eye examinations. Genomic DNA was extracted from peripheral blood according to standard protocols [32].

Parts of the *ABCA*4 gene were cloned from a genomic canine λ-DNA library (λ FIX^®^II Library; host: *E. coli *XL1-Blu MRA (P2) Stratagene, La Jolla, CA, USA) according to the Stratagene standard protocol and described in detail elsewhere [10]. In order to characterize the flanking introns of exon 3 and 6 of the *ABCA*4 gene one isolated λ-clone was directly sequenced with exonic primers specific for exons 3 and 6. The other exon/intron boundaries of the *ABCA*4 gene were analyzed by comparing the mRNA sequence of the canine gene (EMBL accession number AJ784316) to the genomic sequence of the human gene (EMBL accession number AF001945). For SSCP analyses DNA sequences of exons 2, 3, 6, 9–10, 12–13, 21–22, 28–29, 34–35, 39–40 and 45–47 of the *ABCA*4 gene were amplified including adjacent intervening sequences [see Additional file 2].

The canine *CX*36 gene was characterized by sequencing genomic DNA with exonic 'human' primers in conserved regions of the gene. The exon/intron boundaries were analyzed by comparison of this canine genomic sequence with mRNA sequence of the human *CX*36 gene (EMBL accession number NM_020660.1). For SSCP analyses DNA sequences of exon 1 and 2 of the *CX*36 gene were amplified by overlapping PCRs including neighboring intronic sequences [see Additional file 2].

Genomic sequence and exon/intron boundaries of *MERTK *and *RDH*12 genes were determined from dog genome databases (UCSC Genome Bioinformatics website, dog genome sequence as of May 2005) by searching with the human mRNA of the two genes (*MERTK*: EMBL accession number U08023.1; *RDH*12: EMBL accession number BC025724.1). Exons 4, 6–8 and 10 of the *MERTK *gene and all 7 exons of the *RDH*12 gene were screened for mutations by SSCP analyses including flanking intron sequences [see Additional file 2].

All PCRs were performed under standard PCR conditions [10-12] in a thermocycler (Biometra, Goettingen, Germany) with Taq Polymerase (Genecraft, Münster, Germany) and varying concentrations of MgCl_2 _[see Additional file 2]. For SSCP analyses, 0.06 μl of [α^32^P] dCTP (10 mCi/ml) was included. PCR products were digested depending on the lengths of the fragments with different restriction enzymes [see Additional file 2] in order to optimize mutation screening by SSCP analysis [1]. Aliquots of the PCRs (3 μl) were denatured with 7 μl of loading buffer (95% deionised formamide 10 mM NaOH, 20 mM EDTA, 0.06% (w/v) xylene cyanol and 0.06% (w/v) bromphenol blue). The samples were heated to 95°C for 5 min and snap cooled on ice. Aliquots (3 μl) of the samples were separated in two sets of 6% polyacrylamide (acrylamide/bisacrylamide: 19/1) gels, one set with 10% glycerol, another containing 5% glycerol and 1 M urea. Gels were run with 1X TBE buffer at 30–50 W for 3–6 h at 4°C, dried and analyzed by autoradiography. All DNA samples exhibiting band shifts as evidenced by SSCP electrophoresis were purified and cycle sequenced. Sequencing reactions were carried out by the dideoxy chain termination method using the Dyenamic ET Terminator Kit (Amersham Biosciences, Freiburg, Germany) according to the manufacturer's instructions and were run on an automated capillary DNA sequencer (MegaBACE 1000, Amersham Biosciences, Freiburg, Germany).

## Authors' contributions

TL conceived the experimental outline, collected blood samples, conducted the experiments, analyzed the data and drafted the manuscript. SMP, BK and SED carried out parts of the experiments and were involved in analyzing the data. JTE and GD participated in design and coordination of the study and helped to draft the manuscript. All authors read and approved the final manuscript.

## Supplementary Material

Additional file 1 Sequence variations in the *ABCA4*, *CX36*, *MERTK *and *RDH12 *genes in different dog breeds and indirect exclusion of the genes as causing gPRA, respectively. The data provided represent the results of the mutation screening of the candidate genes *ABCA4*, *CX36*, *MERTK *and *RDH12*. Click here for file

Additional file 2 Primers, conditions for PCR amplification and restriction enzymes used before SSCP analyses. The data provided represent the information for PCR amplification and restriction of PCR products which were used in SSCP analyses. Click here for file
