# Supplementary material for: Indirect exclusion of four candidate genes for generalized progressive retinal atrophy in several breeds of dogs
Source: J Negat Results Biomed. 2006 Nov 29;5:19. doi: 10.1186/1477-5751-5-19 (PMC1716180; doi:10.1186/1477-5751-5-19)
Supplement: Additional file 1 — Sequence variations in the ABCA4, CX36, MERTK and RDH12 genes in different dog breeds and indirect exclusion of the genes as causing gPRA, respectively. The data provided represent the results of the mutation screening of the candidate genes ABCA4, CX36, MERTK and RDH12. [file 1477-5751-5-19-S1.doc]

Additional file 1: Sequence variations in the *ABCA*4, *CX*36, *MERTK* and *RDH*12 genes in different dog breeds and indirect exclusion of the genes as causing gPRA, respectively.

| Gene | Location | Sequ. variation | Amino acid ex-change | Breed with sequence variation | | | Breed for which the gene was excluded as causing gPRA |
| --- | --- | --- | --- | --- | --- | --- | --- |
|  |  |  |  | in gPRA affected and unaffected dogs | | in heterozygous state in gPRA affected dogs |  |
| *ABCA*4 | Exon 6 | c.588 A>T | P196P | AT, BDP, Co, GS, Ku, Lo, MP, NF, Ro, Sa, ScT, SD, Sl, TT, WD | AT, BDP, Lo, MP, NF, SD, WD | | AW*, AT*, BDP, CdT*, GR*, JRT*, Ku, Lo, MP*, NF*, Sa, Sal, ScT, SD, Sl, TT, WD* |
|  | Intron 6 | IVS6 +53 G>C | - | Aki, JRT, Ku, Lo, Sa, ScT, Sl, TT | Ku, Lo, Sa, ScT, TT | |  |
|  | Intron 6 | IVS6 +114 A>G | - | Aki, JRT, Ku, Lo, Sa, ScT, Sl, TT | Ku, Lo, Sa, ScT, TT | |  |
|  | Intron 9 | IVS9 +191 C>T | - | BDP, CdT, Lo, MP, NF, Sal, SD, Sl, SS, TT | CdT, Lo, MP, NF, Sal, SD | |  |
|  | Exon 13 | c.1880 A>C | E627A | Aki, AW, Bo, CCR, GR, MP, Ro, ScT, SD, SS, TT, WD | AW, GR, MP, SD, TT, WD | |  |
|  | Intron 21 | IVS21 -19 A>G | - | Lo, Sal, SD, Sl | Lo, Sal, SD, Sl | |  |
|  | Intron 28 | IVS28 +23 A>G | - | Lo, Sa, Sal, Sl | Lo, Sa, Sl | |  |
|  | Intron 28 | IVS28 +157 C>A | - | Lo, Sa, Sal, Sl | Lo, Sa, Sl | |  |
|  | Intron 28 | IVS28 +164G>T | - | Lo, Sa, Sal, Sl | Lo, Sa, Sl | |  |
|  | Intron 28 | IVS28 -218 A>G | - | Lo, Sa, Sal, Sl | Lo, Sa, Sl | |  |
|  | Intron 28 | IVS28 -214 A>G | - | AT, BDP, Co, GR, JRT, GS, MP, Sa, SD, TT, WD | BDP, JRT, Sa, SD, TT, WD | |  |

Additional file 1: Continue.

| Gene | Location | Sequ. variation | Amino acid ex-change | Breed with sequence variation | | | Breed for which the gene was excluded as causing gPRA |
| --- | --- | --- | --- | --- | --- | --- | --- |
|  |  |  |  | in gPRA affected and unaffected dogs | | in heterozygous state in gPRA affected dogs |  |
| ABCA4 | Exon 29 | c.4254 A>G | P1418P | Lo, Sa, Sal, Sl | Lo, Sa, Sl | |  |
|  | Exon 29 | c.4263 G>A | E1421E | Lo, Sa, Sal, Sl | Lo, Sa, Sl | |  |
|  | Exon 29 | c.4272 A>G | A1424A | Lo, Sa, Sal, Sl | Lo, Sa, Sl | |  |
|  | Intron 45 | IVS45 +73 C>G | - | Ku | Ku | |  |
|  | Intron 45 | IVS45 +89 A>G | - | Lo, Ro, Sa, Sal, Sl | Lo, Sa, Sal, Sl | |  |
|  | Intron 46 | IVS46 -21 C>T | - | Lo, Ro, Sal, Sl | Lo, Sal, Sl | |  |
|  | Intron 46 | IVS46 -4 A>G | - | Sa | Sa | |  |
| *CX*36 | Intron 1 | IVS1 +81 A>C | - | CdT, Lo, MP, ScT, Sl, TT, WD | CdT, MP, ScT, Sl, TT, WD | | AT, AW, CdT, Ku, Lo, MP, Sa, Sal, ScT, Sl, TT, WD |
|  | Exon 2 | c.621 T>C | N207N | AW, Sal | AW, Sal | |  |
|  | Exon 2 | c.678 A>G | P226P | Aki, AT, AW, BDP, Co, CdT, CCR, Ku, Lo, MP, NF, Sa, Sal, ScT, Sl, TT, WD | AT, AW, CdT, Ku, Lo, MP, Sa, TT, WD | |  |
| *MERTK* | Exon 4 | c.600 C>T | P200P | BCo, BDP, CdT, Co, GIT, GR, GS, Ku, Lo, MP, NF, Ro, Sa, Sal, Sl, SS, TT, WD | BCo, BDP, CdT, Co, GIT, GR, GS, Ku, Lo, MP, NF, Ro, Sa, Sl, SS, TT | | Aki, AW, AT, BCo, BDP, Bo, Co, CdT, CCR, FCR, GIT, GR, GS, JRT, Ku, Lo, MP, NF, PON, Ro, Sa, Sal, ScT, SD, Sl, SS, TT, WD, Y |
|  | Exon 6 | c.918 G>C | P306P | AT, CdT, GR, MP, NF, PON, SS | AT, MP, NF, SS | |  |

Additional file 1: Continue.

| Gene | Location | Sequ. variation | Amino acid ex-change | Breed with sequence variation | | | Breed for which the gene was excluded as causing gPRA |
| --- | --- | --- | --- | --- | --- | --- | --- |
|  |  |  |  | in gPRA affected and unaffected dogs | | in heterozygous state in gPRA affected dogs |  |
| *MERTK* | Exon 7 | c.1093 C>G | P365A | Aki, AT, BCo, Bo, Co, CdT, GIT, Ku, Lo, MP, Sa, Sal, ScT, Sl, TT, WD, Y | Aki, AT, BCo, Co, CdT, GIT, Ku, Lo, MP, Sa, Sal, Sl, TT, WD | |  |
|  | Intron 7 | IVS7 -53 C>T | - | Aki, AW, AT, BCo, BDP, Bo, Co, CdT, CCR, GIT, GR, JRT, Ku, Lo, MP, Sa, Sal, Sl, TT, WD, Y | Aki, AW, AT, BCo, CdT, CCR, GIT, JRT, Lo, MP, Sa, Sal, Sl, TT, WD | |  |
|  | Intron 7 | IVS7 -13 T>C | - | AT |  | |  |
|  | Exon 8 | c.1209 T>C | I403I | Aki, AW, AT, BCo, BDP, Bo, CdT, CCR, FCR, GIT, GR, GS, Ku, Lo, NF, PON, Ro, Sa, Sal, ScT, SD, Sl, SS, WD, Y | Aki, AW, AT, BCo, BDP, Bo, CdT, CCR, FCR, GIT, GS, Ku, Lo, PON, Ro, Sa, Sal, ScT, SD, Sl, SS, WD, Y | |  |
|  | Exon 10 | c.1458 C>A | T486T | BDP, CCR |  | |  |
| *RDH*12 | Exon 1 | c.48 C>T | Y16Y | CdT |  | | AT, CdT, GIT, GR, GS, Ku, Lo, MP, WD |
|  | Intron 4 | IVS4 +46 T>A | - | AT, Co, CdT, CCR, GIT, GR, GS, Ku, Lo, MP, Ro, WD | AT, CdT, GIT, GR, GS, Ku, Lo, MP, WD | |  |

*Exclusion is likely but not definitive, because there was only one polymorphism identified in heterozygous state in the comparatively large *ABCA4* gene.
