# Supplementary material for: Indirect exclusion of four candidate genes for generalized progressive retinal atrophy in several breeds of dogs
Source: J Negat Results Biomed. 2006 Nov 29;5:19. doi: 10.1186/1477-5751-5-19 (PMC1716180; doi:10.1186/1477-5751-5-19)
Supplement: Additional file 2 — Primers, conditions for PCR amplification and restriction enzymes used before SSCP analyses. The data provided represent the information for PCR amplification and restriction of PCR products which were used in SSCP analyses. [file 1477-5751-5-19-S2.doc]

Additional file 2: Primers, conditions for PCR amplification and restriction enzymes used before SSCP analyses.

| PCR-System | Location | Primer sequence (5’🡪3’) | Fragment size (bp) | Annealing temperature/MgCl_2_ (°C/mM) | Restriction enzyme |
| --- | --- | --- | --- | --- | --- |
| ABCR C 2F | Exon 2 | TTCGCTTTGTGGTGGAACTTG | 135 | 54/2.0 | *-* |
| ABCR C IVS2+R | Intron 2 | CTGGCAATCCTGGAGGTCC |
| ABCR C IVS2-F | Intron 2 | CCAGCAACCAGAAGTGGCA | 257 | 54/1.0 | *HpaII* |
| ABCR C IVS3+R | Intron 3 | TTAGGCTATGGCTTGAACGCA |
| ABCR C IVS5-F | Intron 5 | TCTGTGGGTTAGGACACGGAC | 405 | 53/1.0 | *BshNI* |
| ABCR C IVS6+R | Intron 6 | GGCTCCAAAGACCCAAGGA |
| ABCR H 9F | Exon 9 | TGTAATGCATTGATCCAGAGCC | 437 | 54/1.0 | *BstYI* |
| ABCR C IVS9+R | Intron 9 | GTTCTCTGAATCCTGGCTCCC |
| ABCR C IVS9-F | Intron 9 | AACTCAGGGTTTGTGACAGGA | 202 | 54/1.0 | *-* |
| ABCR H 10R | Exon 10 | CTTTGACAACAGCACACAGATG |
| ABCR H 12F | Exon 12 | TGCTTGGTCCTGGATAAGTTTGAAAG | 240 | 54/3.0 | *NlaIII* |
| ABCR C IVS12+R | Intron 12 | AGGCAATCTTGAAGTCCCTGTGC |
| ABCR C IVS12-F | Intron 12 | TGTGCTTGTGAAGTGCCACTAAACTGT | 250 | 54/1.0 | *NlaIII* |
| ABCR H 13R | Exon 13 | TACCCCTGCTTCGTGGACGATTC |
| ABCR H 21F | Exon 21 | GTGGCTGAGCACATGCTGTTC | 208 | 54/1.0 | *-* |
| ABCR C IVS21+R | Intron 21 | ACACTGGCAGAACGGGAGG |
| ABCR C IVS21-F | Intron 21 | CCCTGGGCCTTTCTCCTC | 203 | 54/2.0 | *BshNI* |

Additional file 2: Continue.

| PCR-System | Location | Primer sequence (5’🡪3’) | Fragment size (bp) | Annealing temperature/MgCl_2_ (°C/mM) | Restriction enzyme |
| --- | --- | --- | --- | --- | --- |
| ABCR H 22R | Exon 22 | CTGCTCCTGAAGTATCGCTCAG |  |  |  |
| ABCR C 28F | Exon 28 | TTGGCTCTGATGCTTTCCATC | 656 | 54/3.0 | *BfaI* |
| ABCR C 29R | Exon 29 | CCTGAAGGAAGAATGGCTTCC |
| ABCR H 34F | Exon 34 | GCCCTATCACTAGAGAGGCCTCTAAAG | 468 | 54/1.5 | *MvaI* |
| ABCR H 35R | Exon 35 | GGAGCAGCTCTCAGAGATTACAGT |
| ABCR H 39F | Exon 39 | ACGCTGCTCAGGTTCAACG | 161 | 54/1.0 | *-* |
| ABCR C IVS39+R | Intron 39 | CCTGTGGAACATGGCCCTAC |
| ABCR C IVS39-F | Intron 39 | CAGCTGGGCCAAATGTTGTA | 205 | 54/1.0 | *-* |
| ABCR C 40R | Exon 40 | CCACTTCTTCCTCACCCGATG |
| ABCR H 45F | Exon 45 | TGCAAACTGGAGTATTAAGAGCCTG | 555 | 54/1.0 | *AvaII* |
| ABCR C IVS45+R | Intron 45 | CCTTGTGCCCATGCAGTCTG |
| ABCR C IVS45-F | Intron 45 | TTGTACATTGTCTGCCAGAGAGCAG | 381 | 54/3.0 | *AluI* |
| ABCR H 47R | Exon 47 | CTTTCGATGTATGGGCACCATTCA |
| CX36 H 1F | Exon 1 | TGGGGGAATGGACCATCTTG | 239 | 54/1.0 | *HaeIII* |
| CX36 C IVS1+R | Intron 1 | GGTACCCGATGCCCAGTGAT |
| CX36 C IVS1-F | Intron 1 | GGCAGGGAAAGGAGGAGG | 220 | 56/1,0 | *-* |
| CX36 H 2R | Exon 2 | CCTGCTATGACCGCGCC |

Additional file 2: Continue.

| PCR-System | Location | Primer sequence (5’🡪3’) | Fragment size (bp) | Annealing temperature/MgCl_2_ (°C/mM) | Restriction enzyme |
| --- | --- | --- | --- | --- | --- |
| CX36 C 2F | Exon 2 | GTTTGTGTGCAACACCTGCAG | 442 | 54/0.75 | *MboII* |
| CX36 H 2R1,5 | Exon 2 | AAGGCATCTCCCGCTTCTACA |
| CX36 H 2F | Exon 2 | CCACACCCATCAGGTCTACGC | 441 | 54/1.0 | *HaeIII* |
| CX36 H 2R2 | Exon 2 | GTCCAGTGACTCTGCCTATGTGTG |
| MERTK C IVS-3F | Intron 3 | AACAGTCTCCATTCCCCTTGG | 287 | 57/3.0 | *HincII* |
| MERTK C IVS+4R | Intron 4 | GAAAACAATAAAGAAATCACACAACTCG |
| MERTK C IVS-5F | Intron 5 | ACTGTGTGTGTTCGGTAGCTGTG | 223 | 57/2.0 | *-* |
| MERTK C IVS+6R | Intron 6 | CAGCCACTTCTCTCTTCATCCTG |
| MERTK C IVS-6F | Intron 6 | CCATGTCTGGCATTCCCAC | 276 | 57/2.0 | *Tru1I* |
| MERTK C IVS+7R | Intron 7 | GGCAATATTTATTCTGAACTCCAGG |
| MERTK C IVS-7F | Intron 7 | CCACAGAATGTTCAAAAACCAAAC | 295 | 57/2.0 | *MboI* |
| MERTK C IVS+8R | Intron 8 | CCTAGGAGACCAGTGAACTCCTTC |
| MERTK C IVS-9F | Intron 9 | TCTTTCCCTATTACAAGCCAACATG | 267 | 57/2,0 | *HincII* |
| MERTK C IVS+10R | Intron 10 | AAACATTTGAAGCTGGGACACTTAC |
| RDH12 C 5’UTR | 5' UTR | GCACAAAGAAGCCAGGAGTTG | 231 | 60/1.5 | - |
| RDH12 C IVS1+R | Intron 1 | CTACGCATATCCCAATCCAGG |
| RDH12 C IVS1-F | Intron 1 | GAAATTTCCCTTTGAGGCTGG | 301 | 58/1.5 | *Bsp143II* |

Additional file 2: Continue.

| PCR-System | Location | Primer sequence (5’🡪3’) | Fragment size (bp) | Annealing temperature/MgCl_2_ (°C/mM) | Restriction enzyme |
| --- | --- | --- | --- | --- | --- |
| RDH12 C IVS2+R | Intron 2 | GGAGGAAAGATAAGGAAATGTGGAC |  |  |  |
| RDH12 C IVS2-F | Intron 2 | GGTTTAGGGATGGCTGGGAG | 332 | 58/1,5 | *HinfI* |
| RDH12 C IVS3+R | Intron 3 | GTGGCACAGGGTGGAACAG |
| RDH12 C IVS3-F | Intron 3 | ACTCAGGCTTGCTACAGGCAG | 352 | 58/1.5 | *NsiI* |
| RDH12 C IVS4+R | Intron 4 | CATCAGGCTTCTTGCAGGG |
| RDH12 C IVS4-F | Intron 4 | GCTTCTACCCCCTGAGCCTG | 307 | 62/1.5 | *Bsp143II* |
| RDH12 C IVS5+R | Intron 5 | GTCTCTACCCTGGCTCTTTCTCC |
| RDH12 C IVS5-F | Intron 5 | CCATTTGTGCATTTTGCTGC | 316 | 56/1.5 | *MboI* |
| RDH12 C IVS6+R | Intron 6 | CAGGTGATGGAGAAAACCAGG |
| RDH12 C IVS6-F | Intron 6 | CAACTTTTCCAGATTGCCACC | 273 | 58/1.5 | *Bsp143II* |
| RDH12 C 3'UTR | 3' UTR | GCCAGCCAGGAGGACAGTC |

The letter ‘C’ after the gene designation in the first column points to canine-specific primer sequences, whereas ‘H’ stands for primers deduced from human DNA sequence.
